# Size-Based Proteomic Signatures of Extracellular Vesicles Derived from Umbilical Cord Mesenchymal Stem Cells Fractionated by EXODUS

**DOI:** 10.3390/ijms27167263

**Published:** 2026-08-14

**Authors:** Shan Wang, Yulin Cao, Yali Yu, Anyuan Zhang, Bianlei Yang, Shumei Xiao, Zhichao Chen, Qiubai Li

**Affiliations:** 1 Department of Rheumatology and Immunology, Union Hospital, Tongji Medical College, Huazhong University of Science and Technology, Wuhan 430022, China; cqwshan@hust.edu.cn (S.W.); yulincao@hust.edu.cn (Y.C.); bianleiyang@hust.edu.cn (B.Y.);; 2 Department of Hematology, Union Hospital, Tongji Medical College, Huazhong University of Science and Technology, Wuhan 430022, China; 3 Hubei Engineering Research Center for Application of Extracellular Vesicles, Hubei University of Science and Technology, Xianning 437100, China

**Keywords:** mesenchymal stem cell, extracellular vesicles, proteome, heterogeneity, size, corona, extracellular matrix

## Abstract

Umbilical cord mesenchymal stem cell-derived extracellular vesicles (UCMSC-EVs) hold strong promise for regenerative medicine, yet their intrinsic size heterogeneity remains a critical barrier to clinical translation, as it obscures molecular and functional specialization within bulk EV preparations. Here, we pioneer the application of the automated EXODUS platform to directly fractionate EVs from cell culture supernatants, resolving bulk UCMSC-EVs into three size-defined subpopulations. By integrating this platform with high-resolution mass spectrometry, we systematically characterize the molecular and functional landscapes of these UCMSC-EV size subpopulations. We demonstrate that EV size is tightly linked to distinct biogenetic origins, biomolecular corona composition, and functional programs: smaller EVs are enriched in exosome-associated proteins, ECM–glycan interfaces, and corona-associated molecules, and preferentially engage endocytosis- and phagosome-related pathways, whereas larger EVs exhibit ectosomal signatures. These findings identify EV size as a critical determinant of molecular architecture and biological function, providing insight into size-dependent EV heterogeneity and informing the rational design and optimization of UCMSC-EV-based therapeutic strategies.

## 1. Introduction

Umbilical cord mesenchymal stem cells (UCMSCs) have attracted considerable attention for their clinical potential in regenerative medicine [1,2]. Increasing evidence suggests that their therapeutic effects are largely mediated by their secreted extracellular vesicles (EVs) [3]. EVs are nanoscale, lipid bilayer-enclosed particles released by virtually all cell types and function as important mediators of intercellular communication [4,5]. They carry diverse molecular cargo, including proteins, lipids, and nucleic acids, and transfer these bioactive components to recipient cells to regulate cellular behavior and physiological states [6]. UCMSC-derived EVs (UCMSC-EVs) are considered the principal paracrine mediators of their parental cells and have been shown to recapitulate many of their therapeutic functions, including immunomodulation [7], angiogenesis regulation [8], inflammation suppression [9], and tissue regeneration [10]. Compared with cell-based therapies, EV-based “cell-free” strategies offer improved safety while maintaining biological activity [11,12].

However, EV populations exhibit pronounced heterogeneity, particularly in size, and this physical diversity directly translates into divergence in molecular composition and biological function [13,14,15,16]. Bulk EV preparations may obscure functionally distinct subpopulations, potentially leading to misleading or averaged biological readouts. Such masking effects may arise when different EV subsets exert divergent or even opposing functions, when redundant functional populations are unevenly represented, or when specific bioactive subtypes are diluted within heterogeneous mixtures [17].

To address this challenge, and to better define the molecular basis of size-dependent EV heterogeneity, this study systematically characterizes UCMSC-EV subpopulations across defined size ranges. Using the automated EXODUS (exosome detection via the ultrafast-isolation system) platform [18], an emerging high-resolution EV fractionation system, EVs can be separated based on physical properties with improved precision and reproducibility. EXODUS is an automated filtration platform that incorporates negative pressure oscillation and double coupled harmonic oscillator-enabled membrane vibration to reduce membrane clogging and enhance EV recovery, allowing for efficient and reproducible EV isolation with high purity. By adjusting nanoporous membranes with defined pore sizes, this device can fractionate EVs based on their physical dimensions. In this study, we adopted pore sizes of 200 nm, 100 nm, and 20 nm to sequentially fractionate UCMSC-EVs into large (200–450 nm), medium (100–200 nm), and small (20–100 nm) subsets. These cutoffs were selected based on the chip specifications, with reference to previous EXODUS-based validations in urine and tear samples [18,19], and are broadly aligned with the MISEV2023 operational definitions of EV subpopulations [20]. Previous studies have demonstrated the applicability of this platform for size-based EV isolation from low-volume clinical biofluids such as urine and tears [18,19]. However, its performance and applicability in processing large-volume cell culture supernatants remain largely unexplored. In this study, we report, for the first time, the successful application of EXODUS to the systematic fractionation of EV subpopulations directly from conditioned UCMSC culture media. Combined with high-resolution mass spectrometry (MS), we isolated size-resolved EV subpopulations, profiled their protein landscapes, and investigated their associated functional signatures. This integrated approach reveals distinct size-dependent molecular architectures and functional biases across EV subsets, providing a framework for understanding how EV size shapes biogenesis, surface organization, and biological activity, and offering a basis for the rational optimization of UCMSC-EV-based therapeutic strategies.

## 2. Results

### 2.1. Identification and Characterization of UCMSCs

**Figure 1:**
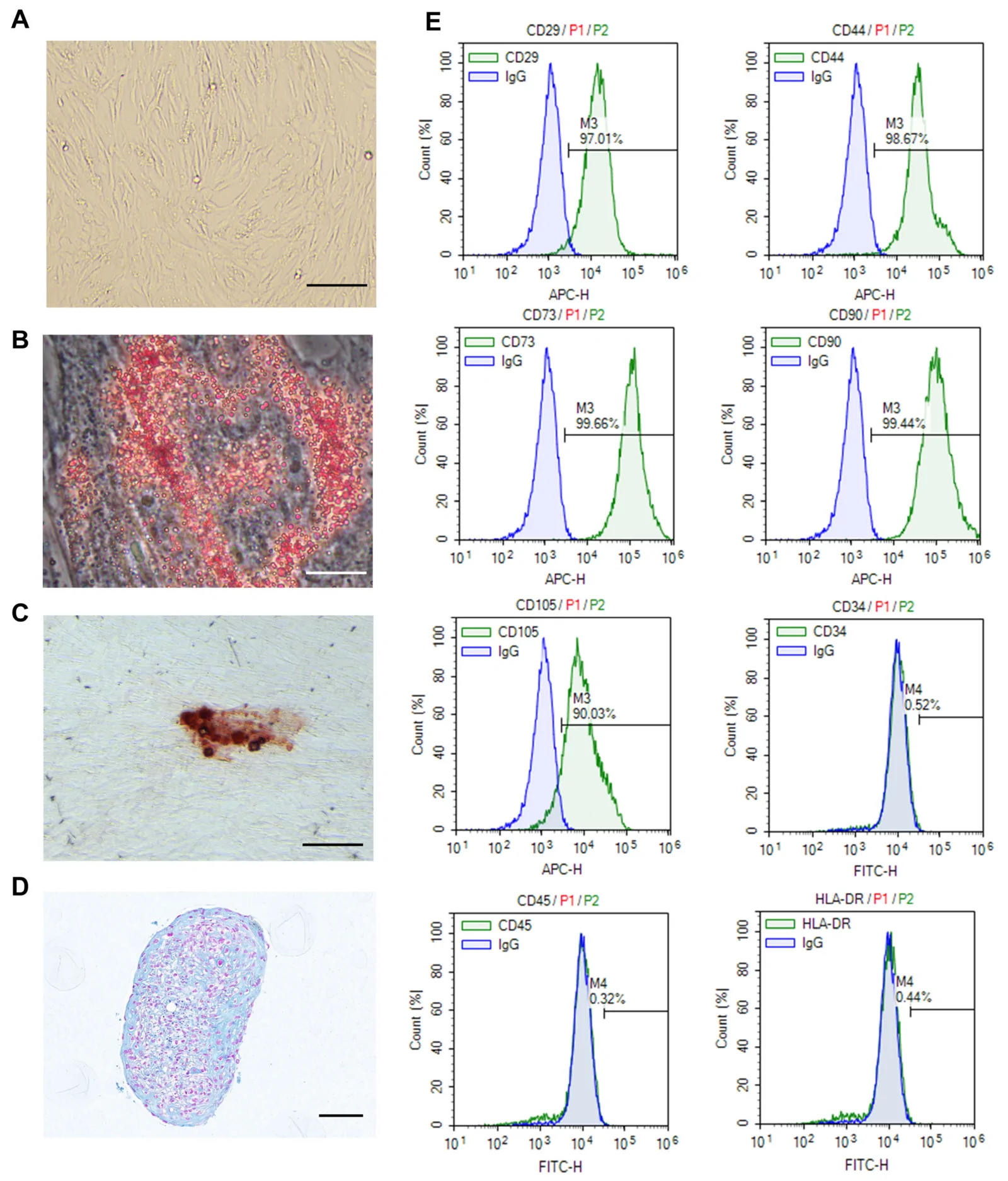
Identification and characterization of UCMSCs. (**A**) The morphology of cultured UCMSCs. Scale bar = 20 μm. (**B**) Adipogenic differentiation of UCMSCs as assessed by Oil Red O staining. Scale bar = 20 μm. (**C**) Osteogenic differentiation of UCMSCs as assessed by Alizarin Red S staining Scale bar = 20 μm. (**D**) Chondrogenic differentiation of UCMSCs as assessed by Alcian Blue staining of sulfated glycosaminoglycans in micromass pellet culture. Scale bar = 100 μm. (**E**) Immunophenotypic profile of UCMSCs determined by flow cytometry.

To ensure the reliability of the downstream EV isolation, we first rigorously isolated and characterized the parental UCMSCs. Morphological observation revealed that the primary cultured UCMSCs readily adhered to the plastic culture flasks. The cells exhibited a typical spindle-shaped, fibroblast-like morphology and assembled into characteristic swirling or whorl-like patterns upon reaching high confluence (Figure 1A). To verify the multipotency of the isolated cells, in vitro directed differentiation assays were performed. Following incubation in specific induction media, the UCMSCs demonstrated robust multi-lineage differentiation potential. Adipogenic differentiation was confirmed after 14 days of induction, characterized by the prominent accumulation of intracellular lipid droplets (Figure 1B). Similarly, the osteogenic capacity of the cells was validated after 21 days of directed culture, as evidenced by the formation of mineralized calcium nodules (Figure 1C). Chondrogenic differentiation was confirmed after 21 days of micromass pellet culture in chondrogenic induction medium, characterized by the robust accumulation of glycosaminoglycans (Figure 1D). Furthermore, the surface immunophenotype of the cultured UCMSCs was quantitatively analyzed using flow cytometry. As illustrated in Figure 1E, the isolated cells robustly expressed classical MSC surface antigens, showing exceptional positivity for CD73 (99.66%), CD90 (99.44%), CD44 (98.67%), CD29 (97.01%), and CD105 (90.03%). Conversely, these cells lacked the expression of hematopoietic and endothelial lineage markers, displaying negligible levels of CD34 (0.52%) and CD45 (0.32%), as well as the major histocompatibility complex class II antigen HLA-DR (0.44%).

Collectively, these morphological, differentiation, and immunophenotypic profiles confirm that the isolated cells are highly pure, biologically functional UCMSCs, thereby establishing a reliable cellular foundation for the subsequent extraction and proteomic profiling of their distinct EV size subsets.

### 2.2. EXODUS-Mediated Size-Based Fractionation of UCMSC-EVs

**Figure 2:**
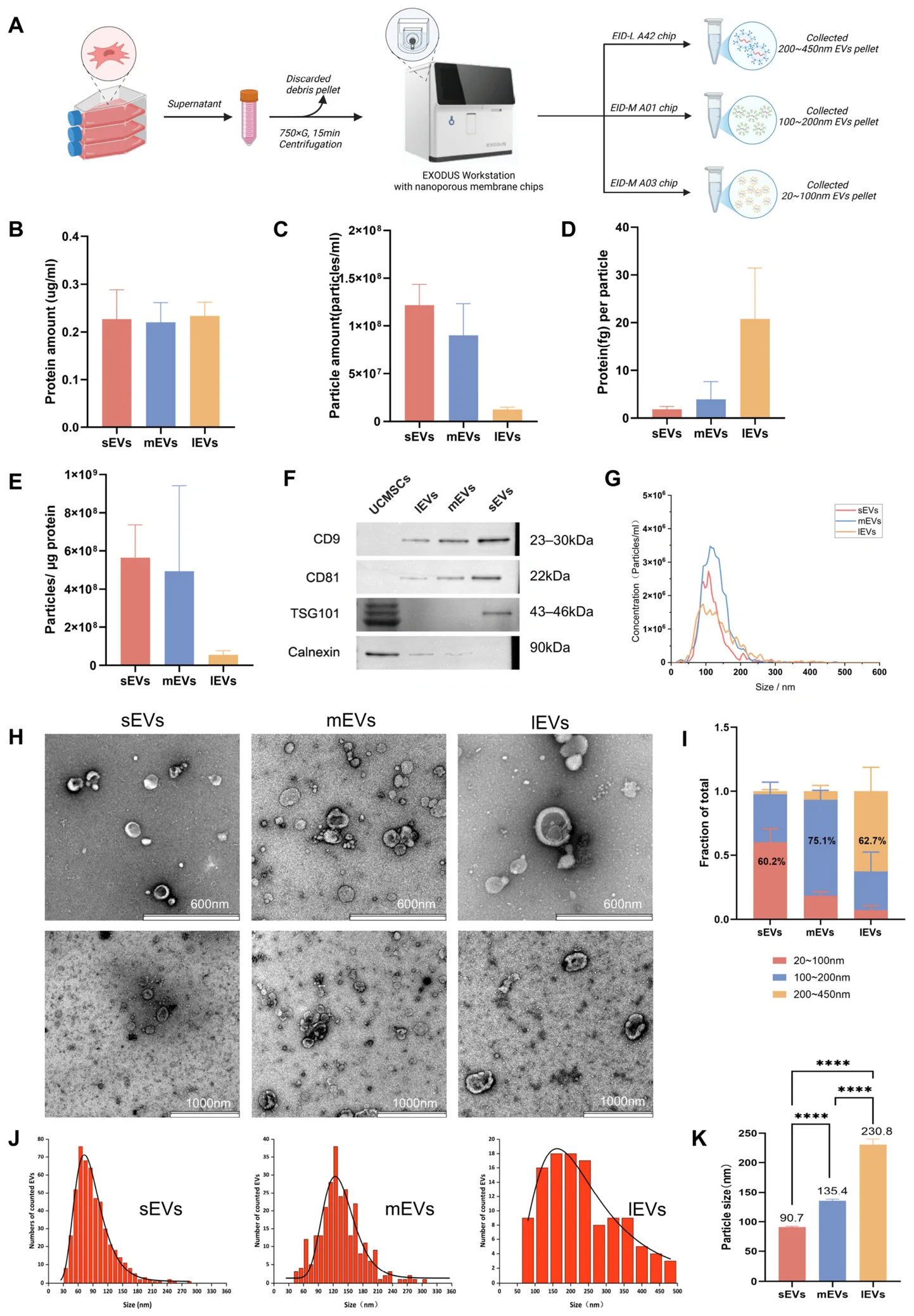
Isolation and characterization of three distinct size subsets of UCMSC-EVs. (**A**) Schematic workflow for isolation and size fractionation of UCMSC-EVs. (**B**,**C**) Protein yield (**B**) and particle concentration (**C**) of the isolated EV subsets (*n* = 3 biological replicates). (**D**) Protein-per-particle value of the isolated EV subsets (*n* = 3 biological replicates). (**E**) Particle-to-protein ratio of the isolated EV subsets (*n* = 3 biological replicates). (**F**) Western blot analysis of canonical EV markers (*n* = 3 biological replicates). (**G**) Size distribution of the EV subsets measured by NTA (*n* = 3 biological replicates). (**H**) Representative TEM images revealing the morphology of the EV subsets (*n* = 3 biological replicates). Scale bars = 600 nm (top row) and 1000 nm (bottom row). (**I**) Quantitative fraction of particles within specific size ranges (20–100 nm, 100–200 nm, and 200–450 nm) for each EV subset, determined by microscopic counting (*n* = 3 biological replicates). (**J**) Size frequency distribution histograms and corresponding fitted curves based on TEM particle counting for each subset. (**K**) Statistical comparison of the average particle size among the three EV subsets. Data are presented as mean ± SD. **** *p* < 0.0001.

We employed the EXODUS platform to isolate and fractionate EVs from the conditioned medium of cultured UCMSCs into three distinct size subpopulations: small EVs (sEVs, 20 to 100 nm), medium EVs (mEVs, 100 to 200 nm), and large EVs (lEVs, 200 to 450 nm). This size-based fractionation was achieved by adapting EXODUS chip membranes with defined pore sizes of 20 nm, 100 nm, and 200 nm (Figure 2A). While the protein yields were similar among the subsets (Figure 2B), the total particle yield was predominantly concentrated in the sEV and mEV fractions (Figure 2C). Notably, the lEV fraction exhibited a higher protein-per-particle value but a lower particle-to-protein ratio compared to the sEV and mEV fractions (Figure 2D,E). The expression of TSG101, CD81, CD9, and calnexin across the different EV subpopulations was assessed by Western blot analysis (Figure 2F). The tetraspanins CD9 and CD81 were detected in all subpopulations. In contrast, the endosomal sorting complex required for transport (ESCRT)-associated protein TSG101 was enriched in the sEV fraction but was barely detectable in mEVs and lEVs, suggesting there are differences in cellular origin among the three EV subpopulations. Calnexin was undetectable in the sEV fraction but showed weak signals in the mEV and lEV fractions. This distribution pattern is consistent with previous reports demonstrating that Calnexin can be detected in larger EV fractions while being absent from small EVs derived from various cell types [21].

We next assessed the size distributions and morphological characteristics of the three UCMSC-EV subpopulations using Nanoparticle Tracking Analysis (NTA) and transmission electron microscopy (TEM). Initial NTA measurements revealed that while the peak modal diameters of the three subsets were relatively clustered—a well-documented phenomenon attributable to the hydrodynamic measurement principles and light-scattering biases inherent to NTA platforms—the overall distribution profiles exhibited clear, subset-specific trends (Figure 2G). Notably, the distribution curves of the mEV and lEV fractions, particularly the lEV fraction, displayed distinctly broader profiles with an extended tail in the larger diameter range (>150 nm). In contrast, the sEV fraction presented a much narrower, tightly restricted distribution peak.

Given that NTA profiles can be confounded by particle hydration layers and Brownian motion dynamics in polydisperse samples, we employed high-resolution TEM to definitively validate the morphological integrity and fractionation efficiency. TEM imaging confirmed the presence of intact, membrane-bound structures with characteristic cup-shaped morphologies across all three groups (Figure 2H). To quantitatively confirm the physical separation, rigorous image analysis and microscopic counting of the TEM micrographs were performed. The statistical evaluation demonstrated a high degree of size-specific enrichment corresponding to the designated subsets (Figure 2I). Specifically, vesicles ranging from 20 to 100 nm constituted the clear majority of the sEV subset, accounting for 60.2% of the total population. Similarly, the mEV subset was highly enriched with particles in the 100 to 200 nm range (75.1%), while larger vesicles spanning 200 to 450 nm were the predominant species within the lEV subset (62.7%). Although minor overlaps between size categories were observed, such distributions are an expected outcome given the continuous physical nature of EV biogenesis. Consistent with these enrichment fractions, the distribution histograms and mean diameters calculated directly from the TEM micrographs revealed a clear, stepwise size separation: the sEVs displayed the smallest mean diameter (90.7 ± 1.6 nm), mEVs showed an intermediate size (135.4 ± 2.8 nm), and lEVs were characterized by the largest vesicular structures (230.8 ± 9.5 nm) (Figure 2J,K).

Taken together, these data confirm that the EXODUS platform successfully fractionated the bulk UCMSC-EVs into structurally distinct small, medium, and large subsets, thereby providing a robust foundation for subsequent proteomic profiling.

### 2.3. UCMSC-EV Size Subpopulations Exhibit Distinct Protein Signatures

**Figure 3:**
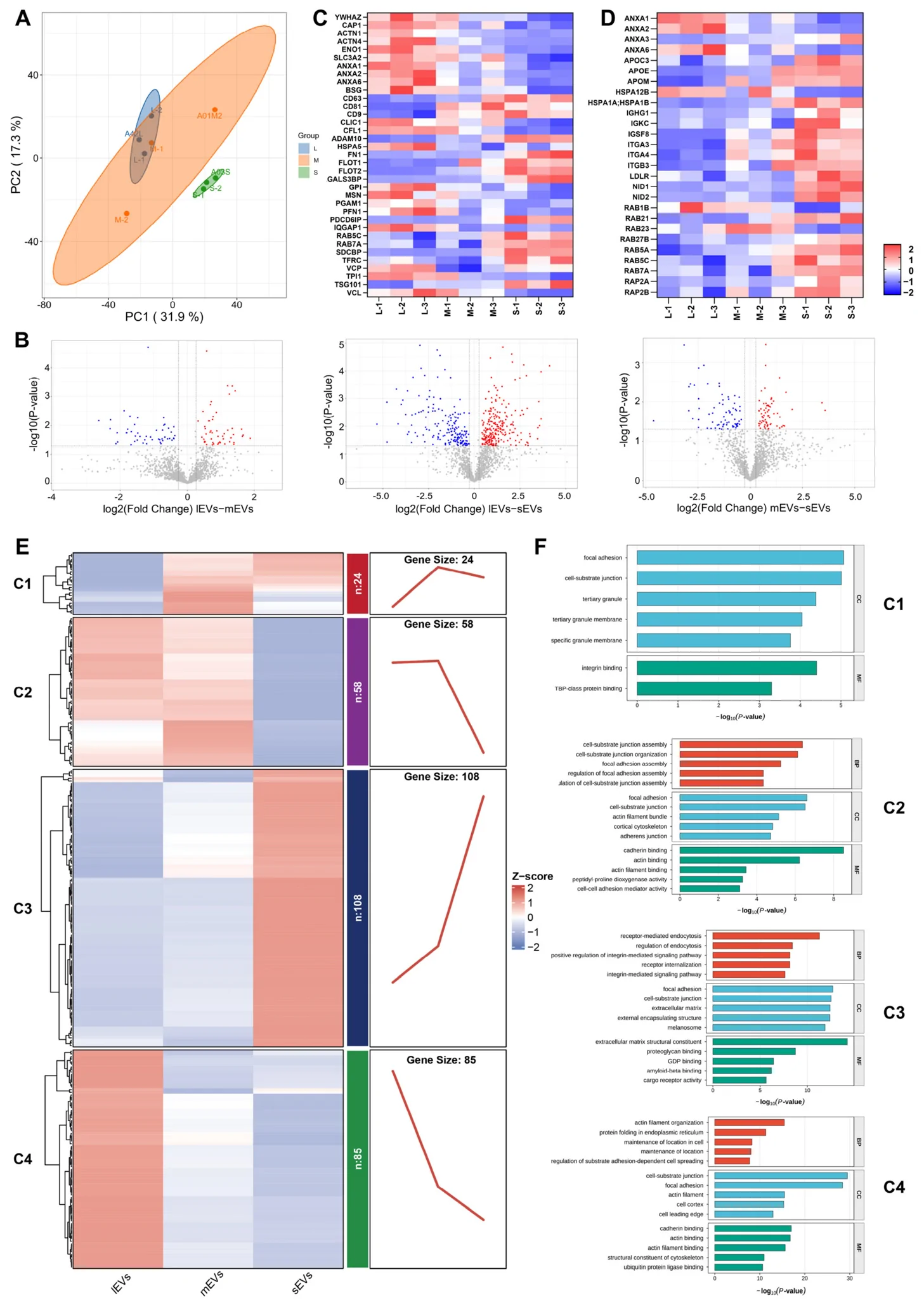
Proteomic profiling reveals distinct protein signatures among the three EV size subsets. (**A**) A principal component analysis based on the MS results. (**B**) Volcano plots showing differential protein expression between the EV subsets: lEVs vs. mEVs (**top**), lEVs vs. sEVs (**middle**), and mEVs vs. sEVs (**bottom**). (**C**,**D**) Heatmaps of individual protein enrichment profiles for canonical EV markers (**C**) and specific protein families (**D**). (**E**) Hierarchical clustering analysis based on the MS results. (**F**) Gene ontology enrichment for respectively cluster 1, 2, 3 and 4.

To delineate the molecular blueprints of the UCMSC-EV size subpopulations, high-resolution mass spectrometry-based proteomic analysis was performed. Principal Component Analysis (PCA) of the quantified proteins (Figure 3A) revealed clear size-dependent spatial segregation. The sEVs formed a highly distinct, tight cluster separated from the larger vesicle populations along PC1 (31.9% variance). While lEV replicates also clustered consistently, the mEV subset exhibited broader dispersion and partially overlapped with the lEVs confidence ellipse. This distribution indicates that sEVs possess a thoroughly unique protein cargo, whereas intermediate mEVs share transitional proteomic characteristics with lEVs.

Pairwise quantitative comparisons were subsequently performed to identify differentially expressed proteins (Figure 3B). The comparison between lEVs and sEVs revealed the most prominent proteomic disparity, encompassing 418 DEPs with 240 proteins up-regulated in lEVs and 178 proteins up-regulated in sEVs. In contrast, the lEVs vs. mEVs comparison yielded the narrowest divergence, with only 51 proteins up-regulated and 48 proteins down-regulated in lEVs, corroborating the compositional similarity observed in the PCA. Finally, the mEVs vs. sEVs comparison identified 68 proteins up-regulated in mEVs and 72 proteins up-regulated in sEVs. Collectively, these data confirm that UCMSC-EV size subsets harbor specialized protein cargos, with sEVs maintaining a distinct molecular signature separate from their larger counterparts.

Closer examination of individual protein expression revealed enrichment of canonical EV markers across the three subpopulations (Figure 3C). The heatmap revealed a size-dependent dichotomy. The sEV fraction was enriched in classical exosome-associated tetraspanins (CD9, CD63, CD81) and essential ESCRT-machinery components (TSG101, SDCBP). Conversely, the lEV fraction was characterized by abundant plasma membrane and cytoskeletal-associated proteins, prominently Annexins (ANXA1, ANXA2, ANXA6), Actinins (ACTN1, ACTN4), Basigin (BSG), and SLC3A2. This compartmentalization supports the paradigm that larger vesicles reflect plasma membrane-derived ectosome characteristics, whereas the smallest vesicles possess classical endosomal origins.

Subsequent analysis of specific protein families revealed that the sEV fraction was enriched in a distinct repertoire of surface integrins (ITGA3, ITGA4, ITGB3) and an extensive suite of Rab GTPases (e.g., RAB5A, RAB5C, RAB7A, RAB27B) (Figure 3D). The specific accumulation of these Rab proteins underscores a highly active, size-specific endosomal sorting and membrane trafficking network intrinsic to sEV biogenesis. Furthermore, particular attention was directed toward proteins frequently recognized as components of the biomolecular corona. Interestingly, the heatmap demonstrated that classical corona-associated apolipoproteins (APOE, APOM, APOC3), alongside immunoglobulin heavy constant gamma (IGHG1), were preferentially enriched in the sEV fraction, maintaining relatively low baseline levels in the larger subsets. This preferential association suggests that the physical dimensions and membrane curvature of EVs may biochemically dictate the formation and composition of their biomolecular corona.

To investigate possible differences in protein ontology among the distinct EV subpopulations, we conducted a clustering analysis that mapped the DEPs into four highly size-dependent dynamic profiles, followed by a Gene Ontology enrichment analysis (Figure 3E,F). Clusters 4 and 3 define the two extremes of the vesicle size spectrum. Proteins in Cluster 4 exhibit a strict, stepwise decrease as vesicle size diminishes, constituting the lEV-specific profile. This lEV-specific profile was enriched in cytoskeletal and plasma membrane-associated terms (e.g., “cell cortex,” “actin filament organization”). Conversely, Cluster 3 maintains low baseline expression in larger vesicles but is drastically upregulated in sEVs. Functionally, the sEV-specific profile shows enrichment for “receptor-mediated endocytosis”, “receptor internalization”, and “extracellular matrix structural constituent”. Bridging the larger subsets, Cluster 2 maintains high abundance in both lEVs and mEVs but experiences abrupt downregulation in sEVs, establishing a clear biochemical boundary separating them from the small fraction. Accordingly, this cluster was enriched in structural and adhesive terms such as “cell–substrate junction assembly,” and “cadherin binding”. Cluster 1 displays an “inverted V-shaped” trajectory peaking specifically in the mEV fraction while remaining at low baseline levels in both lEVs and sEVs. This mEV-specific profile is associated with specialized secretory frames, including “tertiary granule membrane” and “integrin binding.”

### 2.4. Pathway Enrichment Analyses Reveal UCMSC-EVs Display Size-Specific Functionality

**Figure 4:**
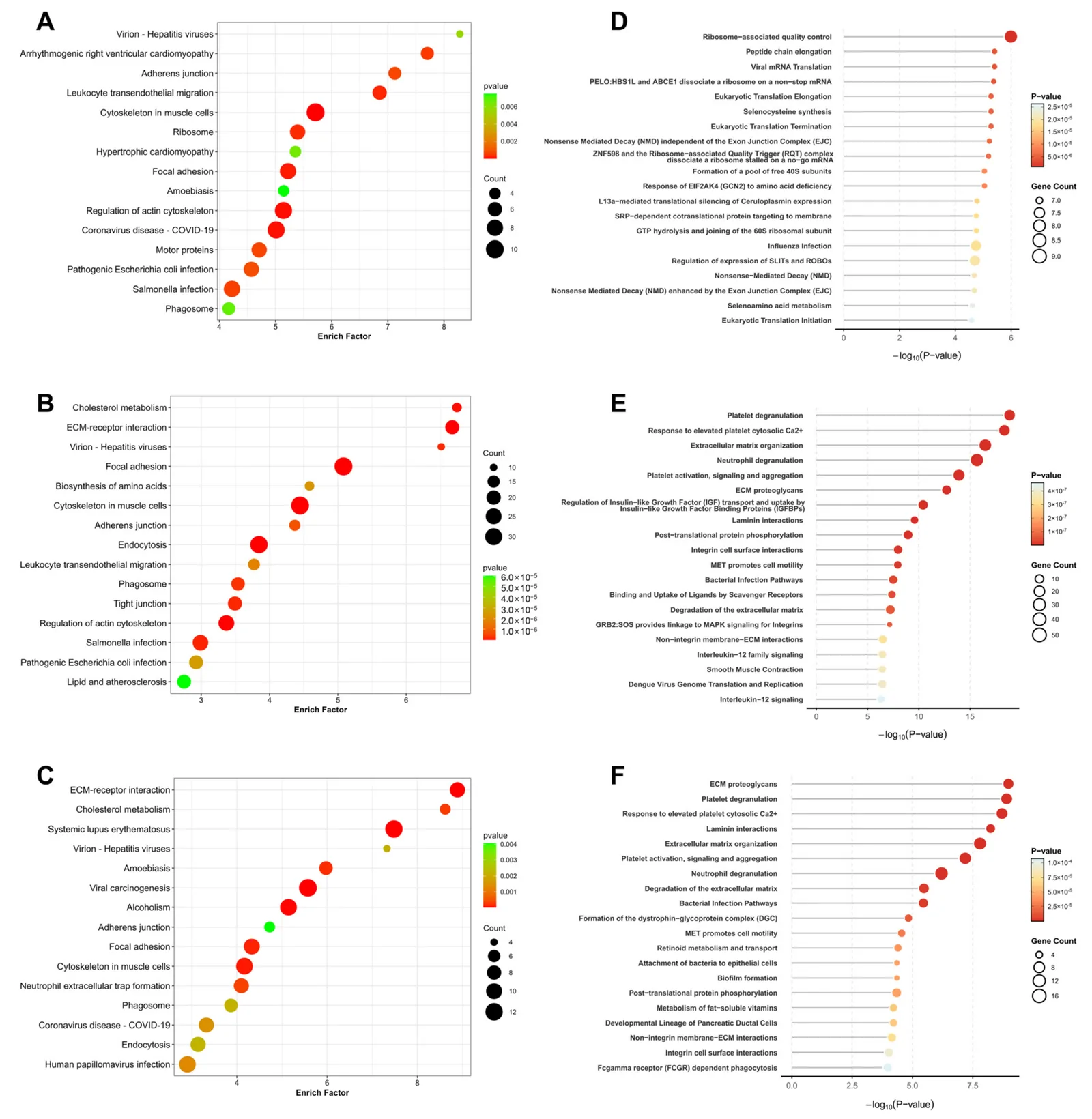
Functional enrichment analysis of the DEPs among the three EV size subsets. (**A**–**C**) KEGG pathway enrichment analysis of the DEPs in the pairwise comparisons: lEVs vs. mEVs (**A**), lEVs vs. sEVs (**B**), and mEVs vs. sEVs (**C**). (**D**–**F**) Reactome pathway enrichment analysis of the DEPs in the pairwise comparisons: lEVs vs. mEVs (**D**), lEVs vs. sEVs (**E**), and mEVs vs. sEVs (**F**).

To further elucidate the systemic biological functions modulated by the UCMSC-EV subpopulations, KEGG pathway enrichment was performed. The proteomic divergence between lEVs and mEVs (Figure 4A) was primarily driven by cytoskeletal and cellular trans-barrier interactions, with significant enrichment in “Leukocyte transendothelial migration” and “Adherens junction,” indicating differing capacities to navigate endothelial barriers. Notably, when comparing sEVs to either of the larger fractions (Figure 4B,C), the functional profiles revealed a shift toward “Cholesterol metabolism,” “Endocytosis,” and “Phagosome.” We further performed Reactome pathway enrichment analysis. Interestingly, the functional divergence between lEVs and mEVs centered around protein synthesis and RNA biology (Figure 4D), dominated by “Ribosome-associated quality control,” “Peptide chain elongation,” and “Eukaryotic Translation Elongation.” This indicates that lEVs package a substantial abundance of macromolecular complexes related to protein translation compared to mEVs. Subsequently, when comparing the larger vesicle fractions to sEVs (Figure 4E,F), the Reactome profiles shifted dramatically toward biological processes associated with extracellular microenvironment interactions and effector cell activation. Among these, the most significantly enriched pathways included “Extracellular matrix organization,” “Integrin cell surface interaction,” and “Neutrophil degranulation.”

### 2.5. WGCNA Identifies Mutually Exclusive Co-Expression Networks Driving UCMSC-EV Size Heterogeneity

**Figure 5:**
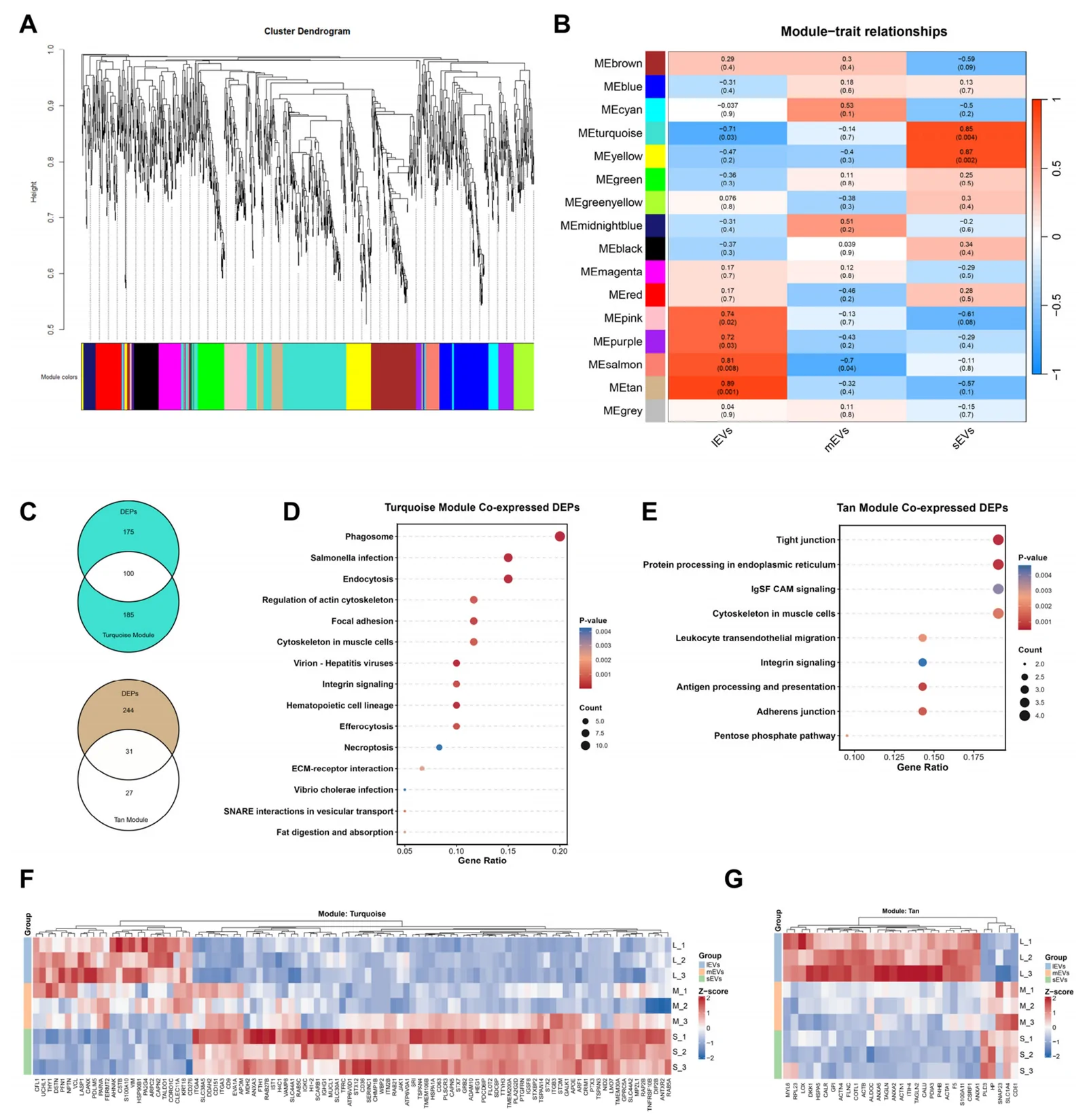
Weighted gene co-expression network analysis (WGCNA) reveals subset-specific protein modules of UCMSC-EVs. (**A**) Hierarchical cluster dendrogram of the identified proteins. (**B**) Heatmap of module-trait relationships illustrating the correlation between the identified protein modules and the three EV size subsets (lEVs, mEVs, and sEVs). (**C**) Venn diagrams displaying the overlap between the DEPs and the proteins within the turquoise module (**top**) and the tan module (**bottom**). (**D**,**E**) Functional pathway enrichment analysis of the overlapping co-expressed DEPs from the turquoise module (**D**) and the tan module (**E**). (**F**,**G**) Expression heatmaps of the hub proteins identified from the turquoise module (**F**) and the tan module (**G**) across the three EV size subsets.

To systematically translate the observed molecular divergences into continuous mathematical correlations and to identify candidate co-expression modules that may drive size-dependent heterogeneity, we performed Weighted Gene Co-expression Network Analysis (WGCNA) to complement our primary differential expression and pathway enrichment findings. By clustering proteins with similar expression trajectories, 16 distinct co-expression modules were identified (Figure 5A). Correlating these module eigengenes with specific EV size traits revealed highly significant modular dichotomies (Figure 5B). Specifically, the Turquoise module exhibited the strongest positive correlation with the sEV subset (r = 0.85, *p* = 0.004) and a negative correlation with lEVs (r = −0.71, *p* = 0.03). Conversely, the Tan module emerged as the critical cluster for larger vesicles, demonstrating a robust positive correlation exclusively with the lEV subset (r = 0.89, *p* = 0.001). To identify the biological drivers within these trait-specific networks, we intersected the module proteins with the previously identified DEPs (Figure 5C). This filtering yielded 100 DEPs confidently mapped to the sEV-associated Turquoise module and 31 DEPs to the lEV-associated Tan module. The top highly connected “hub proteins” from both modules were extracted to evaluate their size-dependent expression patterns (Figure 5F,G). The Turquoise hub proteins maintained severely repressed baseline expression in the larger subsets but were drastically upregulated exclusively in sEVs. Crucially, this mathematically derived sEV hub network prominently featured canonical endosomal markers (CD9, SDCBP), Rab GTPases (RAB27B, RAB5C), specific integrins (ITGA4), and notably, the classical biomolecular corona components (APOE, APOM). Conversely, the Tan hub proteins formed a distinct expression block highly abundant in lEVs, encompassing key plasma membrane and cytoskeletal regulators such as Annexins (ANXA1, ANXA2) and Actinins (ACTN1, ACTN4), which was progressively suppressed as vesicle size decreased. Finally, pathway enrichment of these rigorously filtered co-expressed DEPs mirrored our earlier findings. The co-expressed DEPs from the Turquoise module were enriched in pathways governing vesicle internalization and cytoskeletal dynamics, primarily including “Phagosome,” “Endocytosis,” and “Regulation of actin cytoskeleton” (Figure 5D). On the other hand, the co-expressed DEPs from the Tan module were significantly associated with structural and barrier-modulating pathways, such as “Tight junction,” “Protein processing in endoplasmic reticulum,” and “Leukocyte transendothelial migration.” (Figure 5E) This distinct enrichment signature suggests the lEV payload’s specialization for trans-endothelial communication.

### 2.6. Identification of Core Regulatory Interactomes Driving UCMSC-EV Functional Divergence

**Figure 6:**
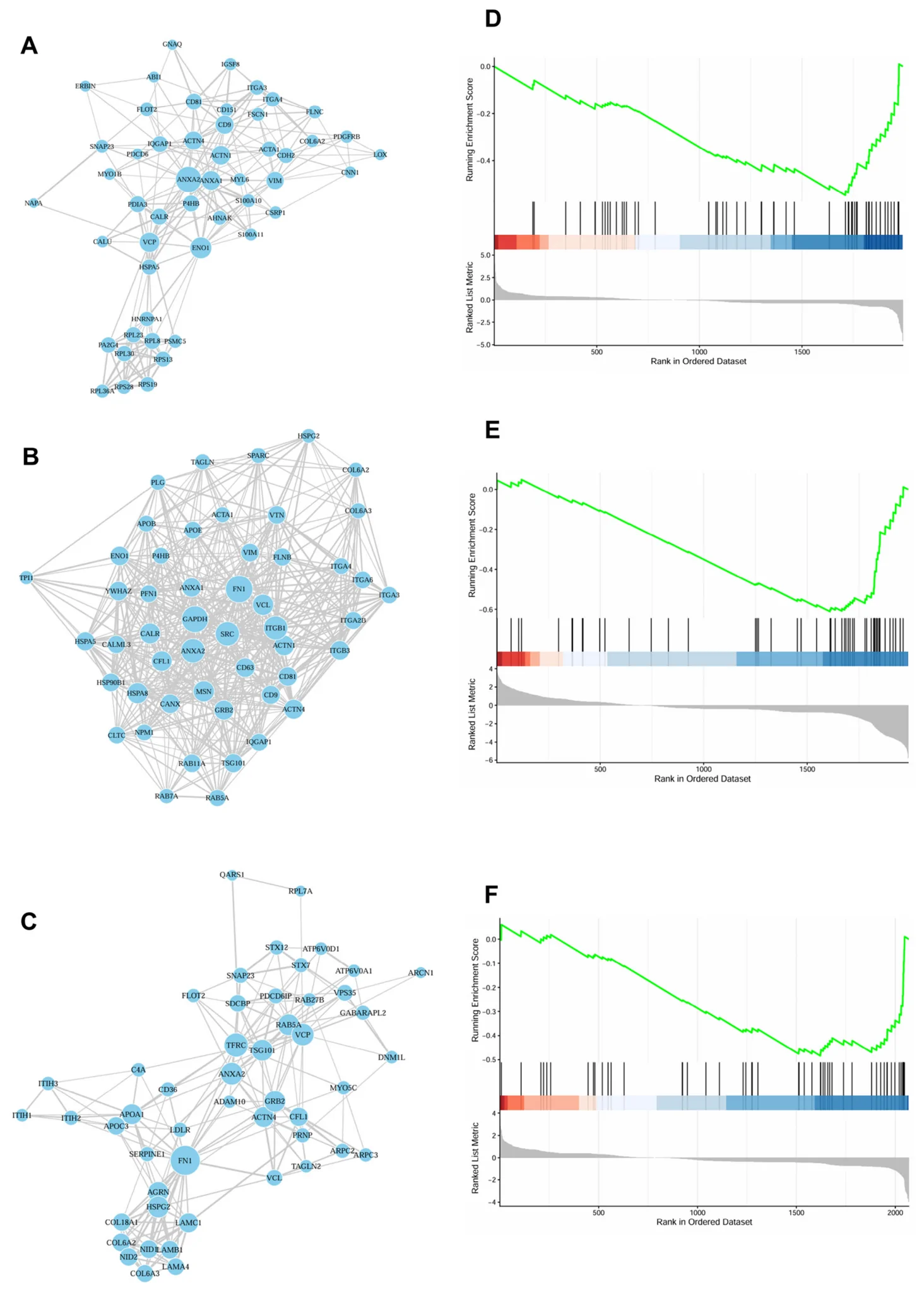
Identification of core regulatory interactomes driving EV size heterogeneity. (**A**–**C**) PPI networks constructed from the top 50 highly connected hub genes identified in the pairwise comparisons: (**A**) lEVs vs. mEVs, (**B**) lEVs vs. sEVs, and (**C**) mEVs vs. sEVs. Node size is proportional to the degree of connectivity within the network. (**D**–**F**) GSEA demonstrating the global expression dynamics of the “ECM–receptor interaction” pathway in (**D**) lEVs vs. mEVs, (**E**) lEVs vs. sEVs, and (**F**) mEVs vs. sEVs.

To pinpoint the critical regulatory hubs driving these functional divergences, Protein–Protein Interaction (PPI) networks were constructed using the top 50 highly connected genes from each pairwise comparison. For the lEV vs. mEV interactome (Figure 6A), the network centered around membrane-cytoskeletal organizers (ANXA1/2, ACTN1/4) interacting with canonical tetraspanins. Strikingly, an independent sub-cluster composed entirely of ribosomal proteins (e.g., RPLP1, RPL8, RPS13) formed at the periphery. This distinct ribosomal hub corroborates, at the protein-interaction level, that the differential packaging of translational machinery primarily segregates the large and medium vesicles.

In contrast, comparing the size extremes (lEV vs. sEV, Figure 6B) revealed a highly integrated network dominated by structural and extracellular matrix (ECM)-interacting proteins. Fibronectin 1 (FN1) and the tyrosine kinase SRC emerged as the paramount central hubs, forming an extensive interaction web with integrins (ITGA3/6, ITGB1), collagens, and heat shock proteins. The mEV vs. sEV comparison (Figure 6C) exhibited a similarly specialized ECM-centric landscape. While FN1 remained a massive central hub, its interacting modules highlighted unique functional segregations: one sub-network was dedicated to ECM organization (laminins, nidogens), while a second module highlighted endosomal sorting and trafficking (TSG101, SDCBP, RAB5C). These networks collectively demonstrate that sEV divergence is fundamentally driven by highly coordinated modules of ECM interactors. Further Gene Set Enrichment Analysis (GSEA) showed consistent results with the PPI findings. The “ECM–receptor interaction” pathway exhibited a robust negative enrichment profile across all three pairwise comparisons (Figure 6D–F). Specifically, the enrichment curves were significantly skewed toward the smaller vesicle subsets in the lEV vs. mEV (NES = −1.93, *p* < 0.001), mEV vs. sEV (NES = −1.76, *p* = 0.002), and lEV vs. sEV (NES = −1.81, *p* < 0.001) comparisons, suggesting that the packaging of protein networks mediating ECM–receptor interactions progressively increases as vesicle size diminishes, culminating in the highest global abundance within the sEV fraction.

## 3. Discussion

UCMSC-EVs have attracted considerable attention for their clinical potential in regenerative medicine because they recapitulate key paracrine functions of their parental cells while avoiding some of the practical and safety limitations associated with live-cell administration. However, intrinsic EV heterogeneity remains a major barrier to their translational application [22,23]. As a fundamental physical property, EV size is a key determinant of molecular composition. Given that variations in cargo abundance and composition can influence biological activity, it is reasonable to infer that EVs derived from the same cellular source but differing in size may exhibit distinct functional properties.

In this study, we employed the automated EXODUS platform to fractionate bulk UCMSC-EVs into three size-defined subpopulations: sEVs (20–100 nm), mEVs (100–200 nm), and lEVs (200–450 nm). Although EXODUS has previously been applied to EV fractionation from clinical biofluids such as urine and tears [18,19], our work represents its first application for the systematic resolution of EV subpopulations directly from conditioned cell culture media. Quantitative morphological assessments confirmed successful size enrichment in each fraction, with particles within the target ranges accounting for 60.2% of sEVs, 75.1% of mEVs, and 62.7% of lEVs, respectively. Consistent with previous reports showing a log-linear decrease in EV concentration with increasing size [24,25,26], we observed the highest particle yield in the sEV fraction, followed by mEVs, with lEVs showing a marked reduction in abundance. This observation also aligns with previous applications of the EXODUS platform, where lEV fractions isolated from tear samples also showed higher protein content and lower particle numbers compared to smaller EV fractions [19]. Although larger EVs possess greater cargo-carrying capacity—for example, a 150 nm vesicle has approximately 25-fold greater surface area and 125-fold greater volume than a 30 nm vesicle, allowing for potentially increased molecular loading [16]—their lower recovery suggests that they may be underrepresented in bulk EV preparations. Consequently, functional readouts derived from bulk EVs are likely dominated by smaller EV populations, which may mask the biological contributions of larger EV subtypes.

By coupling rigorous size-based isolation with high-resolution mass spectrometry, we constructed a size-dependent molecular landscape of UCMSC-EVs. Our proteomic analysis revealed a clear dichotomy in biogenesis-associated signatures across size-defined subpopulations. The sEV fraction was enriched in classical exosome-associated tetraspanins (CD9, CD63, CD81) and core ESCRT-machinery components (TSG101, SDCBP), supporting an endosomal sorting origin for this vesicle population [20,27,28]. In contrast, lEVs showed enrichment of plasma membrane- and cytoskeleton-associated proteins, including annexins and actinins, and lacked detectable ESCRT-machinery protein expression, a profile consistent with direct plasma membrane outward budding characteristic of ectosomes [29]. These protein expression patterns closely resemble those reported in a previous study using a two-step multimodal flow-through chromatography combined with size exclusion chromatography to fractionate cardiac progenitor cell-derived EV subpopulations [13], further supporting the robustness of size-resolved EV heterogeneity across different cell sources and isolation strategies. Importantly, dynamic profiling revealed that mEVs exhibit a distinct protein expression and functional annotation profile that is not simply intermediate between sEVs and lEVs. Rather, this fraction displays unique molecular features, suggesting an independent mode of EV biogenesis and cargo loading, thereby contributing an additional layer to the spectrum of EV heterogeneity.

A striking finding of this study is the size-dependent recruitment of biomolecular corona proteins [30,31,32]. The sEV fraction was prominently enriched in canonical corona-associated apolipoproteins (APOE, APOM, APOC3) as well as immunoglobulins, compared with mEVs and lEVs. These observations are consistent with a recent report showing that small porcine seminal EVs (<200 nm) exhibit a more pronounced protein corona than larger vesicles (>200 nm) under cryogenic electron microscopy [33]. In the context of synthetic nanoparticles, particle size is a well-established determinant of protein corona composition and abundance, largely through its effects on surface area, curvature, and protein-binding properties. However, how EV size regulates corona formation and how such regulation contributes to EV biological function remains insufficiently understood. Our data provide preliminary evidence that EV size influences corona formation, resulting in differential enrichment of specific corona-associated proteins across EV subpopulations. One possible explanation is a threshold effect related to the surface area-to-volume ratio (approximately <180 nm in diameter), whereby smaller vesicles may preferentially present membrane-associated proteins on their surface, thereby facilitating greater corona protein association [34]. Given that surface-associated molecules are increasingly recognized as key determinants of EV function [16,35], these findings suggest that corona composition may represent an additional layer of size-dependent functional regulation. Further studies are needed to elucidate the mechanistic basis of corona-driven functional specialization in EV subpopulations.

Another notable finding of this study is the significant enrichment of ECM proteins, particularly fibronectin (FN1), in the sEV fraction compared with mEVs and lEVs. A potential explanation for this biased distribution lies in the distinct biogenetic origins of EV subpopulations. Fibronectin has been reported to follow an integrin-associated intracellular trafficking pathway, in which it is endocytosed, sorted into multivesicular bodies, and subsequently incorporated onto the surface of exosomes during secretion [36,37]. In this context, exosome-derived sEVs may acquire more fibronectin through this endosomal route, which is not accessible to ectosome-derived lEVs. Beyond individual protein enrichment, FN1 emerged as a central hub in PPI network analysis when comparing sEVs with mEVs and lEVs. This is consistent with the progressive enrichment of ECM–receptor interaction networks as vesicle size decreases, reaching maximal abundance in the sEV fraction. Reactome pathway analysis further confirmed that ECM–glycan interaction pathways are significantly overrepresented in sEVs. In parallel, functional enrichment analysis of both mEV vs. sEV and lEV vs. sEV comparisons revealed a convergent shift toward endocytosis- and phagosome-related pathways. This functional transition may be mechanistically linked to the enriched ECM interface on sEVs. ECM components on EV surfaces have been reported to facilitate vesicle uptake by recipient cells [38,39]. In particular, fibronectin can simultaneously bind to heparan sulfate proteoglycans on both EV and plasma membranes, acting as an adhesion bridge that promotes EV–cell interaction and internalization [40]. Therefore, the enrichment of ECM proteins and ECM glycan-mediated interfaces in sEVs may contribute to their enhanced engagement with endocytic and phagocytic uptake pathways observed in functional profiling.

We acknowledge several limitations in this study. First, the WGCNA was performed on a relatively small sample size (*n* = 9), which is below the recommended minimum of 15 samples for robust network construction. Consequently, we treated WGCNA as an exploratory tool to complement our primary findings rather than as confirmatory analysis. Future studies with larger sample cohorts will be needed to validate the WGCNA-derived module assignments. Second, while this study provides initial evidence for size-dependent corona formation, we acknowledge that definitive validation of bona fide EV corona proteins requires additional experimental approaches. Future studies incorporating immune electron microscopy, confocal imaging, and functional assays will be essential to confirm the size-based EV-surface association of corona proteins and to elucidate their biological significance.

Although the EXODUS platform enabled effective size-based fractionation, EV populations exist along a continuous spectrum, and size-defined fractions may still contain vesicles with heterogeneous origins and properties. Therefore, future studies should integrate physical separation with molecularly targeted approaches, such as immunoaffinity-based isolation, to improve the resolution of EV subpopulation characterization and enable more precise dissection of EV heterogeneity. However, improved isolation specificity often comes at the expense of particle yield. Low recovery remains a major technical challenge, particularly when multiple purification steps are combined. Thus, further methodological optimization is required to better balance selectivity and yield, allowing the acquisition of EV subpopulations with both sufficient purity and adequate quantity for downstream functional and molecular analyses.

## 4. Materials and Methods

### 4.1. Cell Culture

Fresh umbilical cords were obtained from consenting donors who delivered full-term neonates by cesarean section, in accordance with a previously described procedure [41]. Immediately upon delivery, the cord tissues were immersed in sterile phosphate-buffered saline (PBS) supplemented with 100 U/mL penicillin and 100 μg/mL streptomycin (all sourced from Gibco, Grand Island, NY, USA), and transported to the laboratory on ice within 2 to 4 h. Prior to use, the fetal bovine serum (FBS; Gibco) intended for culture was diluted in DMEM-F12 medium and subjected to ultracentrifugation at 100,000× *g* for 18 h at 4 °C using an OPTIMA XPN-100 Ultra-centrifuge (Beckman Coulter, Brea, CA, USA) to deplete bovine-derived EVs. The isolation of mesenchymal stem cells was executed utilizing the tissue explant adherence method. Briefly, the cords were repeatedly washed with pre-chilled PBS to remove residual blood. Umbilical arteries and the vein were meticulously stripped and discarded. The remaining Wharton’s jelly was mechanically minced into fine fragments of approximately 1–2 mm^3^. These explants were uniformly distributed into 10-cm culture dishes and maintained in DMEM-F12 medium (Gibco) enriched with 10% FBS and 1% penicillin-streptomycin. Cultures were incubated at 37 °C in a humidified atmosphere containing 5% CO_2_. The culture medium was carefully replenished every 3 days after an initial 7-day undisturbed attachment period. The cells were passaged upon reaching 80–90% confluence using 0.25% Trypsin-EDTA (Gibco). The UCMSCs used in the experiments were from passages 3 to 5.

### 4.2. Characterization of UCMSCs

The isolated human UCMSCs were cultured on plastic substrates and regularly monitored under an inverted light microscope to verify their adherent growth and characteristic fibroblast-like spindle morphology. For immunophenotypic characterization, UCMSCs were detached using 0.25% Trypsin-EDTA, washed with PBS, and resuspended at a density of 1 × 10^6^ cells/mL. Cell aliquots were incubated in the dark at 4 °C for 30 min with fluorochrome-conjugated monoclonal antibodies against MSC-specific positive surface markers (anti-CD29, anti-CD44, anti-CD73, anti-CD90, and anti-CD105) and negative cocktail markers (anti-CD34, anti-CD45, and anti-HLA-DR) (Abclonal Technology, Wuhan, China). After removing unbound antibodies via sequential washes, the fluorescence profiles were acquired using a flow cytometer (Novocyte, Agilent, Santa Clara, CA, USA) and analyzed with FlowJo software (Version 10.8.1). To assess osteogenic differentiation, UCMSCs at passage 3 were seeded into 6-well plates and stimulated with osteogenic induction medium (HUXUC-90021, OriCell, Cyagen Biosciences, Santa Clara, CA, USA) for 21 days. Extracellular calcium-mineralized nodules were fixed with 4% paraformaldehyde (PFA) and visualized via staining with a 2% Alizarin Red S solution for 10 min. For adipogenic differentiation, confluent cell monolayers were exposed to adipogenic induction medium (HUXUC-90031, OriCell) for 14 days. Intracellular lipid droplet accumulation was verified by fixing the cells with 4% PFA and performing Oil Red O staining. For chondrogenic differentiation, UCMSCs at passage 3 were subjected to micromass pellet culture in chondrogenic induction medium (HUXUC-90041, OriCell) for 21 days. The generated cartilaginous spheroids were harvested, fixed in 4% PFA, embedded in paraffin, and cut into 5 μm thick sections. To confirm the synthesis of cartilage-specific extracellular matrix and the deposition of sulfated glycosaminoglycans, the histological sections were stained with an Alcian Blue solution. All stained samples were observed and imaged using an inverted microscope (CKX41, OLYMPUS, Tokyo, Japan).

### 4.3. Isolation and Size Fractionation of UCMSC-EVs

UCMSC-conditioned medium was collected from passage 3–5 cells at 80–90% confluence, and cleared of cellular debris by centrifugation at 750× *g* for 15 min at 4 °C. UCMSC-EVs were isolated and fractionated from the conditioned medium using the automated EXODUS H-600 device equipped with nanoporous chips of defined pore sizes (all sourced from Shenzhen Huixin Biomedical Technology, Shenzhen, China). Based on the manufacturer’s specifications and previous validation studies using urine and tear samples, chips with pore sizes of 200 nm, 100 nm, and 20 nm were applied to sequentially capture large (200–450 nm), medium (100–200 nm), and small (20–100 nm) EV subpopulations. Briefly, the samples were sequentially passed through 0.8-μm and 0.45-μm membrane filters (Millipore, Burlington, MA, USA) to deplete larger protein aggregates, and then loaded into the sample reservoir of the EXODUS-600 device. Large EVs (200–450 nm) were recovered using the EID-L A42 chip (200-nm pore size). The flow-through was subsequently filtered through a 0.22-μm filter (Millipore) and applied to the EID-M A01 chip (100-nm pore size) for medium EVs (100–200 nm), followed by 0.1-μm filtration (Millipore) and capture on the EID-M A03 chip (20-nm pore size) for small EVs (20–100 nm). For each isolation cycle, parameters including sample ID, processing volume, chip specifications, and separation programs were configured via the EXODUS interface. Upon completion of the automated purification programs, the chips were retrieved, and the enriched EV subsets were carefully resuspended in sterile PBS and stored at −80 °C for subsequent analysis.

### 4.4. Western Blot Analysis

Western blot analysis was performed to validate the presence of canonical vesicular markers. Briefly, UCMSC pellets and purified EV samples were lysed on ice using RIPA buffer supplemented with a 1% protease inhibitor cocktail. Following the determination of protein concentrations via BCA assay kit (P0010, Beyotime Biotechnology, Shanghai, China) according to the manufacturer’s instructions, equal amounts of total protein (20 μg) from each sample were denatured at 95 °C for 5 min in loading buffer. The protein extracts were resolved utilizing 10% sodium dodecyl sulfate-polyacrylamide gel electrophoresis (SDS-PAGE) and electrophoretically transferred onto polyvinylidene fluoride (PVDF) membranes (Bio-Rad, Hercules, CA, USA). To mitigate non-specific binding, the membranes were blocked with 5% non-fat dry milk in Tris-buffered saline containing 0.1% Tween-20 (TBST) for 1 h at room temperature. Subsequently, the blots were incubated overnight at 4 °C with primary antibodies targeting EV-enriched proteins, including anti-CD81, anti-CD9, and anti-Alix, alongside anti-Calnexin (all sourced from Abcam, Cambridge, UK) as a negative control to evaluate cellular organelle contamination in the EV fractions. Following thorough washing steps, the membranes were probed with corresponding horseradish peroxidase (HRP)-conjugated secondary antibodies (Abcam). Immunoreactive bands were visualized using an enhanced chemiluminescence (ECL) detection system (Bio-Rad, Hercules, CA, USA).

### 4.5. Nanoparticle Tracking Analysis

To characterize the particle yield of the EV preparations, nanoparticle tracking analysis was performed using a ZetaView PMX-120 analyzer (Particle Metrix, Neubiberg, Germany). The instrument was calibrated prior to sample analysis using known standard polystyrene nanoparticles (100 nm, Thermo Fisher, Waltham, MA, USA). Prior to NTA measurement, each EV sample was diluted with PBS to achieve a concentration within the instrument’s optimal detection range (1~9 × 10^7^ particles/mL), following the manufacturer’s recommendations to ensure accurate and reliable particle counting. Measurements were conducted in scatter mode using a 488 nm laser source at ambient room temperature. The analytical protocol consisted of recording video files across 11 distinct positions within the measurement cell, capturing two cycles per position. The camera sensitivity and shutter parameters were empirically optimized and locked at 80 and 100, respectively, across all measurements to ensure data comparability. The resulting Brownian motion trajectories were analyzed utilizing the ZetaView software (version 8.05.12) to extrapolate both the total particle concentration and the particle size profile.

### 4.6. Transmission Electron Microscopy

Morphological characterization and single-particle sizing of the fractionated EV subsets were performed utilizing a Hitachi HT7800 (Tokyo, Japan) transmission electron microscope. Briefly, a 10 μL aliquot of the purified EV suspension was deposited onto glow-discharged, formvar-carbon-coated copper grids and allowed to adsorb for 5 min at room temperature. Following the removal of excess liquid via capillary action using filter paper, the adherent vesicles were negatively stained with 2% (*w*/*v*) aqueous uranyl acetate for 1 min. The prepared grids were subsequently air-dried under ambient conditions and examined using a Hitachi HT7800 transmission electron microscope operating at an accelerating voltage of 100 kV. For size distribution analysis and statistical particle counting, multiple distinct fields of view were captured randomly. The resulting micrographs were processed using ImageJ software (version 1.54r, NIH, Bethesda, MD, USA), where the diameters of individual vesicular structures exhibiting the characteristic cup-shaped morphology were manually measured to construct a comprehensive size distribution profile.

### 4.7. Proteomic Sample Pretreatment

Proteomic characterization of the different subpopulations was achieved by mass spectrometry analysis of three biological replicates for each UCMSC-EV subpopulation (sEVs, mEVs, lEVs). An equal volume of protein lysis buffer (7 M urea, 2% SDS, supplemented with 2× Protease Inhibitor Cocktail prior to use) was added to the samples. The mixture was sonicated on ice using an ultrasonic cell disruptor (2 s on, 5 s off) for a total of 1 min, followed by lysis on ice for 2 h. The lysate was then centrifuged at 13,000 rpm for 20 min at 4 °C, and the supernatant was transferred to a fresh 1.5 mL Eppendorf tube. Protein concentration was determined using BCA assay.

Based on the measured protein concentration, aliquots containing 40 μg of protein were taken and adjusted to an equal volume using the lysis buffer. Dithiothreitol (DTT) was added to the protein solution to a final concentration of 10 mM, and the mixture was vortexed and incubated at 37 °C for 1 h. Iodoacetamide (IAA) was subsequently added at a DTT:IAA volume ratio of 1:5, thoroughly mixed, and incubated for 40 min in the dark. A five-fold volume of precipitation reagent was added, and the proteins were allowed to precipitate for 1 h. The mixture was centrifuged at 13,000 rpm for 1 h at 4 °C, and the supernatant was discarded. The protein pellet was washed with 1 mL of 100% acetone, centrifuged at 13,000 rpm for 30 min, and the supernatant was discarded; this washing step was repeated once. The pellet was then air-dried at room temperature for 10 min. Trypsin was added, and the proteins were digested overnight at 37 °C.

The dried peptides obtained after centrifugal concentration were desalted using a MonoSpin desalting column and dried again prior to mass spectrometry analysis. The desalting procedure was performed as follows: The dried peptide mixture was dissolved in a 0.1% trifluoroacetic acid (TFA) solution. The column was equilibrated with a 0.1% TFA solution. The reconstituted sample was loaded onto the desalting column and centrifuged. The column was washed with a 0.1% TFA solution. Peptides were eluted by adding a 50% ACN solution followed by centrifugation, and the eluate was collected in a fresh EP tube. The eluted solution was centrifugally concentrated to dryness to remove the ACN.

### 4.8. Mass Spectrometry

Each sample was separated using an ES906 C18 analytical column (PepMap™ Neo UHPLC, 150 µm × 15 cm, 2 µm, Thermo Fisher Scientific, Waltham, MA, USA). Mobile phase A consisted of a 0.1% formic acid aqueous solution, and mobile phase B consisted of 0.1% formic acid in an 84% ACN aqueous solution. Separation was achieved using the following gradient elution program at a flow rate of 2.5 µL/min: 96% mobile phase A for 0–4 min, 75% mobile phase A for 4–5.8 min, 65% mobile phase A for 5.8–6.2 min, and 1% mobile phase A for 6.2–6.9 min. The liquid chromatography system was coupled online to an Orbitrap Astral mass spectrometer (Thermo Fisher Scientific) for detection. The separated peptides were ionized via an electrospray ionization source and directly introduced into the mass spectrometer for data-independent acquisition (DIA) analysis. The ion spray voltage was set to 2.0 kV, and the ion transfer tube temperature was maintained at 290 °C. The mass spectrometer was operated in DIA mode with the following parameters: full MS1 scan range from 380 to 980 *m*/*z*, MS1 resolution of 240,000 (at 200 *m*/*z*), automatic gain control target of 500%, precursor isolation window of 2 Th, 300 DIA windows, and normalized collision energy of 25%. The MS2 scan range was 150–2000 *m*/*z* with a maximum injection time of 3 ms.

For protein identification and quantitative profiling, the raw data were processed utilizing DIA-NN software (version 1.9.2). Database searching was performed against the SwissProt reference database restricted to the Homo sapiens taxonomy. Trypsin was specified as the proteolytic enzyme, allowing for a maximum of two missed cleavages. During the search, carbamidomethylation of cysteine was set as a fixed modification, whereas oxidation of methionine and protein N-terminal acetylation were designated as variable modifications. Spectral matching accuracy was further optimized employing TIMScore based on three-dimensional vectorization. To ensure high-confidence dataset generation, the false discovery rate was strictly constrained to ≤1% at the protein level. Label-free quantification was conducted at the MS2 level, where peptide abundance was determined by integrating the peak areas of their corresponding MS2 fragment ions. Total protein abundance was subsequently computed by aggregating the quantitative values of all matched constituent peptides.

### 4.9. Differential Expression Analysis

The quantitative dataset was subjected to a missing value imputation protocol, wherein null values were replaced with half of the absolute minimum quantitative value observed within the entire detection matrix. Pairwise comparisons across the distinct groups were subsequently executed. The relative expression ratio, defined as the fold change (FC), was derived by comparing the mean normalized signal intensities of the constituent samples per group. The statistical divergence between cohorts was assessed via Student’s *t*-tests. A rigorous dual-filtering paradigm was applied to define the differentially expressed proteins (DEPs); specifically, only targeted proteins demonstrating a statistically significant variance (*p* < 0.05) coupled with an expression fold change of ≥1.2 or ≤0.8333 were retained for subsequent bioinformatic analyses.

### 4.10. Proteomic Clustering and Kinetic Trend Profiling

The mean expression levels of DEPs across the EV subpopulations were isolated for pattern clustering. To normalize the protein abundance profiles, a row-wise Z-score transformation was directly executed on the expression matrix. A K-means clustering algorithm (k = 4) was then implemented to stratify the distinct protein expression modalities based on numerical trajectories. Spatial visualization of these clusters was achieved through a comprehensive heatmap rendered via the R package (version 2.28.0) ComplexHeatmap. To systematically characterize the dynamic fluctuations in protein abundance relative to vesicle dimensions, the mean expression kinetics of each cluster were mathematically computed and graphically represented using trend line plots. The resulting cluster-specific protein cohorts were subsequently extracted to facilitate downstream functional annotation.

### 4.11. Functional Enrichment Analysis

Gene Ontology (GO), Kyoto Encyclopedia of Genes and Genomes (KEGG), and Reactome pathway enrichment analyses, were conducted using the R packages clusterProfiler and ReactomePA. Gene Set Enrichment Analysis (GSEA) was performed on the fully pre-ranked proteomic dataset ((sign(log2FC) × (−log10P)) against human KEGG datasets sourced from msigdbr. A *p* value < 0.05 was considered statistically significant.

### 4.12. Weighted Gene Co-Expression Network Analysis (WGCNA)

The WGCNA R package was employed to build a weighted co-expression network based on the proteomic profiles of all samples (*n* = 9, comprising three EV subpopulations × three biological replicates). To ensure a scale-free network model, the soft-thresholding parameter was established at β = 8 following network topology analysis. Proteins were clustered into distinct modules utilizing the one-step blockwiseModules algorithm, applying a minimum module size of 30 and a merge cut height of 0.25 to consolidate closely related modules. To assess the association between the generated protein modules and the targeted subsets, the distinct vesicle size fractions were encoded into a binary trait matrix. The correlations between the module eigengenes and these phenotypic parameters were then computed using Pearson’s method. To elucidate the biological pathways enriched within these clusters, the overlapping targets between the significant modules and the previously identified DEPs were isolated. These intersecting datasets were subsequently subjected to KEGG functional enrichment analysis utilizing the clusterProfiler tool.

### 4.13. Protein–Protein Interaction (PPI) Network Analysis

A topological protein–protein interaction (PPI) network was developed to evaluate the complex physical and functional associations between the subset-specific proteins. Based on the interaction scores exported from the STRING database (version 12.0), and only those with a combined confidence score ≥ 0.4 were retained for network construction. An undirected network architecture was assembled using the R package igraph. The relative importance of individual proteins within the network was quantitatively assessed by computing their nodal degree. In each experimental group, the top 50 nodes demonstrating the highest degree centrality were identified as critical hub proteins. Ultimately, the dense subnetworks formed by these pivotal targets were isolated and graphically rendered by applying the Fruchterman–Reingold force-directed layout algorithm.

### 4.14. Statistical Analysis

Statistical analysis was performed using GraphPad Prism 8 (GraphPad, San Diego, CA, USA). Statistical significance was calculated by Student’s *t* test and one-way analysis of variance (ANOVA). The data are presented as the mean ± standard error. *p* ≤ 0.05 was considered statistically significant.

## 5. Conclusions

In this study, we systematically resolved UCMSC-EVs into size-defined subpopulations using the automated EXODUS platform and constructed a comprehensive molecular and functional landscape across EV sizes. Our findings demonstrate that EVs exhibit marked size-dependent heterogeneity at multiple levels, including biogenesis-associated protein signatures, biomolecular corona composition, and functional pathway enrichment. Collectively, our results indicate that EV size is a key determinant of molecular composition and biological function, and that bulk EV preparations likely mask functionally distinct subpopulations. A more refined understanding of size-dependent EV heterogeneity, particularly in relation to corona formation and ECM-mediated interactions, may provide important insights for improving EV-based therapeutic strategies.

## Data Availability

The original contributions presented in this study are included in the article. Further inquiries can be directed to the corresponding authors.
